# The Head and Neck Squamous Cell Carcinoma Microenvironment as a Potential Target for Cancer Therapy

**DOI:** 10.3390/cancers11040440

**Published:** 2019-03-28

**Authors:** Jan Plzák, Jan Bouček, Veronika Bandúrová, Michal Kolář, Miluše Hradilová, Pavol Szabo, Lukáš Lacina, Martin Chovanec, Karel Smetana

**Affiliations:** 1 Department of Otorhinolaryngology, Head and Neck Surgery, 1st Faculty of Medicine, Charles University, Prague 15006, Czech Republic; jan.plzak@lf1.cuni.cz (J.P.); jan.boucek@lf1.cuni.cz (J.B.); veronika.bandurova@lf1.cuni.cz (V.B.); 2 Institute of Anatomy, 1st Faculty of Medicine, Charles University, Prague 12800, Czech Republic; pavol.szabo@lf1.cuni.cz (P.S.); lukas.lacina@lf1.cuni.cz (L.L.); 3 Institute of Molecular Genetics, Czech Academy of Sciences, Prague 14220, Czech Republic; kolarmi@img.cas.cz (M.K.); miluse.hradilova@img.cas.cz (M.H.); 4 BIOCEV, 1st Faculty of Medicine, Charles University, Vestec 25250, Czech Republic; 5 Department of Dermatovenerology, 1st Faculty of Medicine, Charles University, Prague 12808, Czech Republic; 6 Department of Otorhinolaryngology, 3rd Faculty of Medicine, Charles University, Prague 10034, Czech Republic; martin.chovanec@lf3.cuni.cz

**Keywords:** cancer, cancer microenvironment, cancer ecosystem, cancer-associated fibroblast, extracellular matrix, cytokine, IL-6, tumour-associated macrophages, cancer therapy

## Abstract

Similarly to other types of malignant tumours, the incidence of head and neck cancer is increasing globally. It is frequently associated with smoking and alcohol abuse, and in a broader sense also with prolonged exposure to these factors during ageing. A higher incidence of tumours observed in younger populations without a history of alcohol and tobacco abuse may be due to HPV infection. Malignant tumours form an intricate ecosystem of cancer cells, fibroblasts, blood/lymphatic capillaries and infiltrating immune cells. This dynamic system, the tumour microenvironment, has a significant impact on the biological properties of cancer cells. The microenvironment participates in the control of local aggressiveness of cancer cells, their growth, and their consequent migration to lymph nodes and distant organs during metastatic spread. In cancers originating from squamous epithelium, a similarity was demonstrated between the cancer microenvironment and healing wounds. In this review, we focus on the specificity of the microenvironment of head and neck cancer with emphasis on the mechanism of intercellular crosstalk manipulation for potential therapeutic application.

## 1. Epidemiology of Head and Neck Squamous Cell Cancer

The incidence of malignant diseases is increasing worldwide [1]. The cause of this dismal trend is being extensively investigated, with an unhealthy lifestyle and environmental pollution being blamed for a large part of it. Besides that, population ageing is associated with age-dependent and gradual loss of capacity of the gene repair machinery. The rapid ageing of the population dependent on a high level of widely accessible medical care (for example in western/central Europe) is accompanied by an accumulation of age-related health disorders including cancer [2]. 

Head and neck squamous cell carcinoma (HNSCC) follows these general oncological trends. Approximately 600,000 patients worldwide suffer from HNSCC [3,4]. Tobacco smoking and alcohol consumption are traditionally linked to this cancer type formation. More recently, human papillomaviruses (HPV) have been proved as an important aetiological factor in some patients without the history of alcohol and tobacco abuse [5]. Interestingly, HPV-positive tumours seem to have different epidemiologic and clinical characteristics, and they also differ in the molecular mechanisms driving their progression. This is reflected in the better treatment response and higher survival rates compared to the HPV-negative tumours [6]. 

## 2. Tumour as A Complex Ecosystem Supporting Function of the Cancer Cells and Cancer Stem Cells

**Figure 1:**
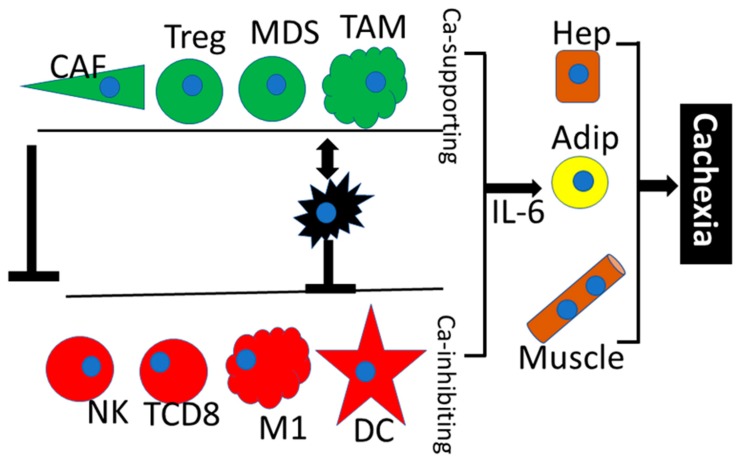
The cancer ecosystem. This figure shows the interaction between cancer cells (black), cancer growth-supporting cells (green) such as cancer-associated fibroblasts (CAF), T-regulatory lymphocytes (Treg), myeloid-derived suppressors (MDS), tumour-associated macrophages (TAM) and cancer growth-inhibiting cells (red) such as natural killer (NK) cells CD8^+^T lymphocytes, M1 macrophages and dendritic cells (DC). The activity of the cells with anti-cancer effect is inhibited both by cancer cells and by cancer-stimulating cells. At an advanced stage of the disease, the entire cancer ecosystem controls the metabolism of the patient mainly through hepatocytes (Hep), adipocytes (Adip) and striated muscle cells (Muscle) by production of factors such as IL-6 that induce failure of the organism and cachexia.

Cells in multicellular organisms must closely collaborate both at the tissue and at the organism level and thus form an analogue of an ecological ecosystem. Intimate communication between cancer cells and non-cancerous cell populations within the tumour also resembles a complicated ecosystem [7] (Figure 1). From this point of view, the malignant tumour can be interpreted as an aberrant organ with specific intrinsic regulation and systemic effect on the function of the whole organism [8]. Cancer cells interact with non-cancerous members within their ecosystem directly (physically by cell-to-cell contacts) as well as indirectly via soluble bioactive molecules (growth factors, cytokines and chemokines).

**Figure 2:**
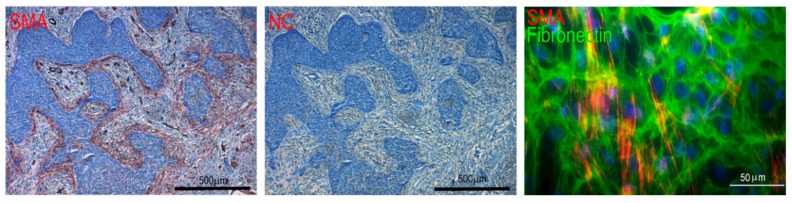
Expression of α-smooth muscle actin (SMA) in basal cell carcinoma and CAF. The figure demonstrates SMA-positive stroma (red) and negative control (NC). Cultured CAF from the human basal cell carcinoma visualised by detection of α-smooth muscle actin (red signal) in the extracellular matrix rich in fibronectin (green signal). Nuclei are counterstained by DAPI (blue signal). The bar represents 500 and 50 μm, respectively. Specimens were prepared in the authors’ laboratory.

A similarity between a healing wound and tumour stroma was proposed by Dvorak more than thirty years ago [9]. Later, comparative research confirmed that cancer ecosystems of various tumours share similar features to the wound at different levels of description [10]. Both ecosystems, i.e., granulation tissue of the wound and cancer stroma, contain activated fibroblasts, myofibroblasts, that express α-smooth muscle actin (SMA) (Figure 2) and fibroblast activation protein (FAP) [10,11]. Multiple types of immune cells also frequently reside in both healing wound and tumour tissue. Their role in cancer biology is surprisingly multifaceted and cell type-specific. 

Among its other functions, the immune system is responsible for accurate identification and elimination of malignant cells. However, this usually requires proper orchestration of several cell types controlling the immune response checkpoints. Under certain circumstances, the malignant cells are recognised by the immune cells as “self”; therefore, tolerogenic signals prevail and effectors, e.g., cytotoxic lymphocytes, do not eliminate malignant cells. Functions of CD8^+^ lymphocytes, NK cells and M1 polarised macrophages are inhibited, while the activity of tumour-supporting immune cells populations such as Treg lymphocytes, myeloid-derived suppressor cells and tumour-associated macrophages is stimulated [12]. 

In general, the cancer microenvironment represents a potent immunosuppressive milieu that is important for the progression of cancer itself. To revert this tolerance, increased expression of cancer-specific antigens might be used to activate the specific anti-tumour immunity. 

### 2.1. Cancer-Associated Fibroblasts (CAF)

In the usual context of acute wounds, myofibroblasts are responsible for the control of wound contraction. Mechanistically, the consequent approximation of wound edges can facilitate rapid reepithelization. Myofibroblasts are also a hallmark of cancer stroma. However, a purely mechanistic explanation for their occurrence at this site is not straightforward. In both systems, myofibroblasts most likely originate from the local mesenchymal cells or mesenchymal stem cells [13]. However, other cell types, including the hypothetical source in cancer cells *per se* after epithelial-mesenchymal transition, cannot be fully excluded as the source cells [14,15,16], assuming CAF derived from the cancer cells would carry the same genetic alterations or HPV-16 oncogenes. In vitro experiments demonstrated that lung epithelial cells transfected with HPV-16 genes E6 and E7 acquired the mesenchymal phenotype and exhibited remarkable biological effects on co-cultured keratinocytes that are phenotypically similar to cancer cells [17]. However, other experiments demonstrated that origination of CAF from cancer cells is not too probable [14].

Concerning the mechanisms of CAF transformation from their potential precursors, growth factors such as TGF-β1/3 and their downstream signalling targets represent widely accepted candidates [18]. These cytokines trigger a complex signalling programme leading to a specific gene transcription profile. The TGF-β influence can be further corroborated via endogenous lectin galectin-1 [19,20]. The TGF-β signalling has been described as context-dependent, and the effect in the context of tumour microenvironment can be highly variable. Furthermore, fibroblasts in the vicinity of a growing malignant cell clone are not uniform [21,22] and represent a heterogeneous pool of cells. Thus, only a limited subpopulation of these cells would be transformed into SMA-producing myofibroblasts [23]. Monitoring their proportion can even be employed in diagnostics, as it influences the disease-free interval and patient survival [24].

**Figure 3:**
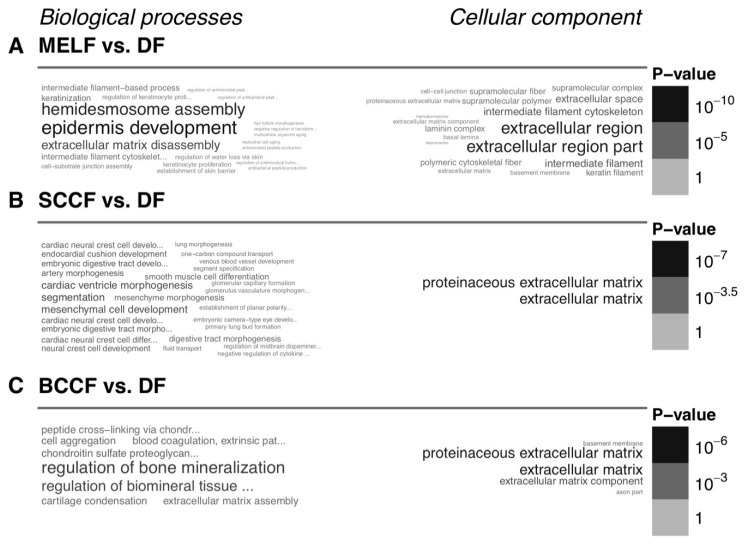
Functional enrichment of the genes differentially expressed between CAF and normal fibroblasts. This figure shows the gene set enrichment analysis using the Gene Ontology terms, where differences between CAF isolated from a skin metastasis of melanoma (MELF, **A**), squamous cell carcinoma (SCCF, **B**) and basal cell carcinoma (BCCF, **C**), and normal dermal fibroblasts from healthy donors (DF, **A**–**C**) are demonstrated. The figure is based on the authors’ data.

Structural and functional differences between CAF and normal fibroblasts represent a very important topic in cancer biology, which has direct implications in clinical oncology. Detailed knowledge of these differences would allow us to control and mitigate their pro-proliferative properties. Available data suggest that CAF broadly differ in expression of hundreds of genes from normal fibroblasts. Differentially regulated genes affect multiple cellular functions. Functional enrichment analysis using the gene ontology (GO) terms indicates that, not surprisingly, the changes in gene expression concentrate in GO terms related to extracellular matrix and developmental processes. These differences from normal fibroblasts are apparent for CAF prepared from melanoma (Figure 3A), squamous cell carcinoma (Figure 3B) and cutaneous basal cell carcinoma (Figure 3C).

**Figure 4:**
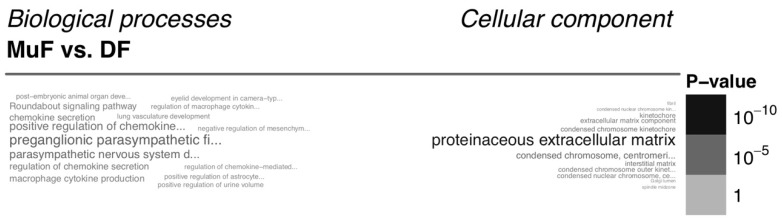
Functional enrichment of the genes differentially expressed between mucous and dermal fibroblasts. This figure shows the gene set enrichment analysis using the Gene Ontology terms, showing the difference between normal mucosa fibroblasts from the oral cavity (MuF) and facial skin (DF). The figure is based on the authors’ data.

On the other hand, normal tissue fibroblasts from different body sites also broadly differ, e.g., normal fibroblasts from oral mucosa differ from the fibroblasts prepared from the skin (Figure 4). Clear differences between normal fibroblasts from the head and body and between fibroblasts of young and elderly patients were also observed [25].

When used as a model of the tumour microenvironment, CAF are biologically active on normal non-cancerous epithelial cells and support cancer cells. Surprisingly, their activity is not cancer type-specific, which suggests that CAF do not use tumour type-restricted mechanisms. For example, CAF isolated from melanoma or basal cell carcinoma influence the phenotype and migratory activity of cells of both breast cancer and glioblastoma [26,27]. However, the in vitro experimental setting used in the cited studies is not fully supported by evidence based on tumour tissue sample analyses, and therefore the results need further validation [28,29,30]. Recently, the epigenetic mechanisms controlling the CAF function within the tumour niche have been identified [30,31]. From the functional point of view, the principal interest of CAF investigation was traditionally oriented on synthesis and turnover of extracellular matrix and production of bioactive factors.

The existence of cancer stem cells and their role was well documented in many types of malignant tumours including HNSCC [32,33]. Their direct identification is complicated because no single specific and robust marker has been determined so far. Their identification is therefore based on combinations of several putative stem cell markers. This panel includes markers such as CD44, CD117 and CD133 [34]. Although stem cell identification is not easy and routinely accessible, their targeting can positively influence the radiosensitivity of HNSCC [35]. The *bona fide* stem cells exhibit unique properties such as slow proliferation rate and long label retention. Rapid transport of xenobiotics from the cytoplasm is another typical functional feature of stem cells. This ability can improve their survival after exposure to toxic agents, e.g., chemotherapy. This phenomenon is a base for the development of critical features of malignancies such as multidrug resistance and long-lasting stabilisation in the stage of minimal residual disease [36].

The proper function of stem cells including cancer stem cells is dependent on their microenvironment. This niche is necessary for the maintenance of their stemness [37,38]. It was shown earlier that local fibroblasts represent a key factor in normal and cancerous tissue models. In these experiments, employment of the normal dermal fibroblasts, as well as CAF, was critical for sustainable maintenance of the stem cell-like phenotype of a side population prepared from the HNSCC FaDu cell line [39]. CAF seem to improve the stem cell properties of a pool of HNSCC cancer stem cells via Wnt-dependent signalling (predominantly Wnt 3a and Wnt 16) [40]. This observation indicates the importance of cancer microenvironment in the biology and clinical properties of cancers, including their stem cell activity.

### 2.2. Extracellular Matrix (ECM) and Cancer

Numerous cell types including CAF produce ECM. ECM represents a fundamental structural component of any normal or pathological tissue. Besides the structural aspects of ECM, it also confers signalling activity, which is decoded by the surrounding cells including cancer cells. The composition of ECM in malignant tumours influences self-sufficient cell growth, insensitivity to growth inhibitors, unlimited replicative potential, angiogenesis and metastatic behaviour. These properties can be modulated directly via specific receptors such as integrins, or by formation of deposits of growth factors or cytokines immobilised to ECM scaffolds [41]. The pattern of ECM in distinct tissues and tumours is not static but highly dynamic. Numerous proteolytic enzymes achieve equilibrium of ECM production and resorption with distinct specificity for different ECM components. This is e.g., essential in the control of the mesenchymal type of cancer cell migration [42]. In addition to protein molecules, complex carbohydrates such as proteoglycans and glycosaminoglycans are present in the ECM, and their importance shall be mentioned [43]. The outstanding number of individual ECM molecules relevant to cancer biology exceeds the scope of this review. However, periostin and tenascin-C should be mentioned here, as they represent the hot topic of ECM research in cancer biology. Nevertheless, the interpretation of the findings is frequently problematic and cancer type-specific [44,45,46,47]. Particular attention is dedicated to endogenous lectins such as galectins and their relation to ECM and cancer progression [48,49]. The similarity between cancer and wound healing microenvironment including ECM expression is remarkable [37,50]. ECM significantly influences not only tumour growth, but also migration of cancer cells and metastatic spread via production of numerous types of collagens and noncollagenous proteins such as tenascin-C, different variants of fibronectin and proteoglycans [51,52].

### 2.3. Growth Factors, Cytokines and Chemokines

As summarised earlier, CAF produce numerous growth factors, cytokines and chemokines—crucial elements of the CAF-cancer cell crosstalk [12,19,50]. As the CAF-cancer cell interaction is not cancer type specific, the existence of some general factors that mediate the interaction may be expected. One of the leading candidates is IL-6 [53]. Although it is generally considered to be an inflammation-initiating molecule, its activity is much broader. IL-6 has a pleiotropic effect, it is elevated in the serum of cancer patients, and a systemic impact of IL-6 on the patient’s organism should be expected [39,54,55]. Increased IL-6 levels in the patients’ sera are indeed associated with dramatic changes in the metabolism of cancer patients because it influences the metabolism of adipocytes, hepatocytes and striated muscle cells, and eventually induces cancer cachexia and wasting [56,57,58]. Moreover, IL-6 crosses the blood-brain barrier, negatively influencing the food intake and participating in depressive syndromes [59,60,61]. The direct effect of IL-6 on cancer cells and other elements of the cancer ecosystem is dependent on the type of activated IL-6 receptor, which exists in membrane-bound and soluble forms. Finally, IL-6 closely collaborates with pro-inflammatory chemokine IL-8. Both factors participate in the control of the migratory activity of cancer cells [62,63,64].

**Figure 5:**
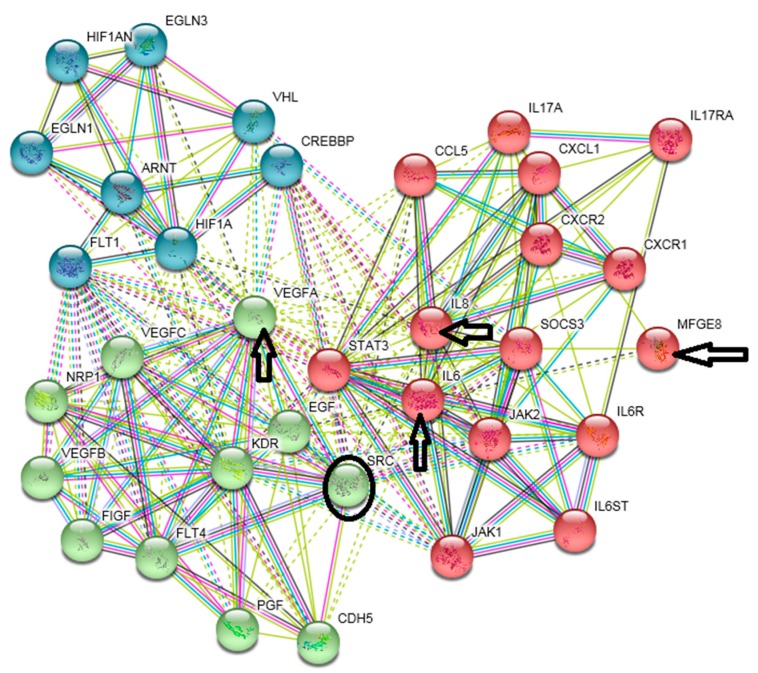
Combinatorial targeting of several signalling pathways. The figure shows the protein interaction analysis (https://string-db.org/) of IL-6, IL-8, VEGF-A and MFGE-8 (indicated by arrows). The circle delineates the SRC protein. Green nodes denote proteins associated with vascularisation, red nodes denote proteins of IL-6/STAT-3 and IL-8 signalling, and blue nodes denote proteins associated with resistance to hypoxia. The links in the diagram indicate experimental or literature association of the proteins. Links in the solid (or dashed) line indicate interactions within (or between) signalling pathways. MFGE-8 also plays a role in the interaction between cancer cells and tumour-associated macrophages (TAM).

Although entirely conclusive results are not yet available, this evidence suggests the IL-6/JAK/STAT3 pathway as a potentially useful target for cancer treatment [65,66]. Perhaps a combination of IL-6 axis blockade combined with targeting of other pathways, such as IL-8 and CXCL-1 axes, may be beneficial [67]. Similarly, blockade of both IL-6 and PD-L1 inhibits growth of hepatocellular carcinoma in the mouse model [68]. Genomic and bioinformatic analyses of cells prepared from basal/squamous cell carcinoma, carcinoma of breast and melanoma demonstrated that simultaneous targeting of IL-6, VEGF-A and MFGE-8 (lactadherin), optionally with inhibition of IL-8, seems to be beneficial in cancer therapy [69]. This combination influences the IL-6-STAT-3 axis, tumour vascularisation and resistance to hypoxia. It may also affect the SRC protein (Figure 5). The combination demonstrates that simultaneous focusing on multiple therapeutic targets can minimise the risk of compensatory bypass of a targeted pathway.

### 2.4. Tumour-Associated Macrophages (TAMs)

TAMs represent another essential component of the cancer microenvironment. Macrophages are recruited to the tumour site by CCL-2-4, CCL-5, -7, -8, -12, VEGF-A, PDGF, M-CSF and IL-10 [70,71,72]. The macrophage pool in the tissue is heterogeneous, with the model of polarisation to M1 and M2 having gained much attention in recent years. To introduce this concept briefly, M1 macrophages collaborate with Th1 lymphocytes in the response to pathogens [73,74]. M2-polarised macrophages are involved in the Th2 immune response, and under “normal” conditions play an important role in the wound repair and tissue remodelling. TAMs share certain properties of both M1- and M2-polarised macrophages.

In general, TAMs have anti-inflammatory properties that both directly and indirectly stimulate cancer cells, tumour growth and metastatic spread. TAMs further contribute to the tumour-supporting microenvironment, e.g., by promoting cancer vascularisation [75]. The main role in the TAM-cancer cell crosstalk is played by production of factors such as MMPs, IL-1β, IL-10, VEGF, PDGF, TGF-β1, MFGE-8, CCL-17, -22, arginase-I and galectin-3 [70]. Concerning potential anti-cancer therapy, emphasis is usually oriented on reduction of the number of TAMs and their controlled repolarisation to M1 macrophages, respectively [72,76,77]. 

### 2.5. Natural Killer (NK) Cells

NK cells represent another leading effector element of the anti-tumour immune response. However, cancer cells and their ecosystem [21] can seriously suppress the NK cell activity. Cancer cells that combine low expression of antigen-presenting MHC-I molecules and high expression of PD-L1 significantly suppress the anti-cancer response by NK elements. Targeting PD1 and PD-L1 both on NK cells and on T lymphocytes represents an excellent strategy for many sensitive tumours [76]. Combination of anti-PD-1/PD-L1 therapy with targeting other receptors such as lectin-like inhibitory receptor NKG2A or EGFR seems to be promising for therapy of some malignancies via activation of NK cells and T lymphocytes [78,79].

### 2.6. Professional Dendritic Cells and CD8^+^ T Lymphocytes

After processing of cancer-specific antigen, dendritic cells activate CD8^+^ lymphocytes. Their function seems to be strongly influenced by the cancer microenvironment that can attenuate the CD-8-dependent immune response to cancer and induce tumour tolerance [79]. Dendritic cells can be prepared in vitro from blood mononuclear cells [80] and used in immunotherapy of tumours via activation of CD8^+^ T lymphocytes [81]. CD8^+^ T lymphocytes represent the main component of anti-cancer immunity. They are activated by dendritic cells with processed tumour-specific antigens [82]. This mechanism is employed in anti-cancer therapy by vaccines prepared from dendritic cells [83]. Their function is positively influenced by IL-2 [84], which has the potential for anti-cancer therapy [85]. Furthermore, therapy combining enhanced activity of CD8^+^ T lymphocytes with other approaches such as anti-PD1/PD-L1 seems to be a perspective for the treatment of resistant tumours [86]. Concerning the target of anti-cancer immunity in HNSCC, the protein exhibiting genetic alterations, aberrantly expressed proteins such is MAGE-A4 antigen normally expressed in testicular cells or virus proteins were discussed [87,88].

### 2.7. Treg Lymphocytes

Treg (FOXP3^+^ CD25^+^CD4^+^) are a critical population for induction of immune tolerance. They also have an inhibitory role in anti-cancer immunity. On the other hand, Treg lymphocytes attenuate chronic inflammation and consequently inhibit tumour initiation related to inflammation [89]. They are strongly attracted to the tumour sites. Depletion of Treg lymphocytes in combination with immune checkpoint inhibitors, such as an antibody against CTLA-4, represent prospective anti-cancer therapy [90].

### 2.8. Myeloid-Derived Suppressor Cells (MDSC)

MDSC represent a highly heterogeneous population. These non-matured myeloid cells have a profound immunosuppressive effect that can stimulate tumour growth. Their positive effect on tumour vascularisation and metastatic spread has been reported [91]. Recent studies demonstrate that neutrophil leukocytes can play a similar role in cancer ecosystems [92]. 

## 3. Exosomes as Important Messengers of the Intercellular Crosstalk in the Cancer Microenvironment

The intercellular crosstalk between cancer cells themselves and between cancer cells and other cell types of the cancer ecosystem is mediated by direct intercellular contacts or indirectly via paracrine secretion of growth factors/cytokines/chemokines. Beside these classical concepts, recent studies have demonstrated that extracellular vesicles can also carry the information necessary for the cell-cell interaction [93]. According to their size and appearance, these vesicles can be classified into the subsets of exosomes (30–150 nm), microvesicles (500–1000 nm) and apoptotic bodies (1000–5000 nm). Exosomes are formed from the endocytic compartment via multivesicular bodies; microvesicles originate by blebbing [94,95]. Exosomes have recently gained much attention in cancer biology as their surface contains many receptors and ligands important for the interaction with cancer cells and other cells of the cancer ecosystem. Nucleic acids (DNA, mRNA, miRNA) and numerous proteins, including growth factors and proteases, are present in their lumen [95]. This cargo makes exosomes capable of influencing gene expression in acceptor cells by transfer of bioactive molecules including gene delivery. Exosomes produced by CAF influence the viability, proliferation and epithelial to mesenchymal transition of cancer cells [96,97]. Cancer cells also produce exosomes that facilitate transition of fibroblasts to CAF [98]. Cells from the HNSCC ecosystem produce exosomes that suppress the activity of anti-cancer lymphocytes and negatively influence the therapy of patients [99]. Their elimination from the patient circulation or preparation of engineered exosomes with anti-cancer activity may have an excellent perspective in future tumour therapy as a tool for manipulation of the cancer microenvironment [100]. 

## 4. Specificity of the HNSCC Microenvironment

The previously described data have general validity for many cancers, including HNSCC. The concept of so-called ‘field cancerization’ indeed belongs to paradigmatic features of HNSCC. The entire epithelial lining of the oral cavity is exposed during the lifetime to the same harmful agents. Thus, we can expect that large areas of the mucosa, if not its entire surface, shares similar genetic alterations. However, clinically apparent tumours arise only in specific regions [101,102,103]. Based on this observation, we can hypothesise about the driving stimuli of such localised progression. It is likely that the diversity of the microenvironment also plays a critical role in the field cancerization. Here, the role of fibroblasts and their potential may be expected [104]. 

The whole ecosystem of HNSCC comprises paracrine and local signalling and mutual crosstalk of cells in the local tumour microenvironment and distal signalling by IL-6 to other parts of the body, e.g., fat tissue, muscles and liver [105]. Indeed, mesenchymal cells including fibroblasts interact with epithelial cells during the developmental processes, e.g., in tooth formation in the prenatal period. This set of developmental events represents a specific morphogenetic programme that can be interpreted as tissue memory [106]. Of note, certain signalling cascades involved in the normal epithelial-mesenchymal interactions during development are also activated in cancerogenesis [107]. Genomic analysis has also demonstrated the expression profile of histologically normal tissue margin of surgical resection to be more similar to cancer than to the healthy tissue [108]. This observation urges for further research because it can change our understanding of radicality in oncologic surgery. 

**Figure 6:**
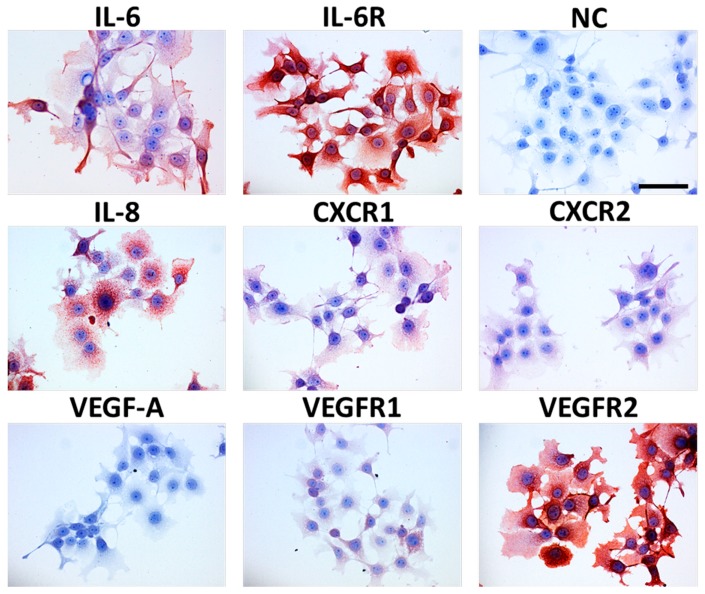
This figure shows HNSCC cell line FaDu from hypopharynx cancer with detected IL-6, IL-8 and VEGF-A and their receptors. NC is a negative control. Nuclei are counterstained with haematoxylin; the bar represents 50 μm. Immunocytochemistry performed in the authors´ laboratory.

Many factors produced by cancer cells as well as other elements of the ecosystem have remarkable biological activity and influence the clinical behaviour of a tumour, as reviewed by Peltanova and coworkers [109]. These factors can be potential targets of anti-cancer therapy. To study the microenvironmental factors in HNSCC, CAF serve as a suitable surrogate as they exhibit a broad spectrum of biological activity on normal keratinocytes in co-cultures. Normal primary keratinocytes consequently acquire an activated phenotype, including markers of epithelial-mesenchymal transition such as co-expression of vimentin and keratins and also transcription factor Snail [110,111]. CAF prepared from HNSCC differ transcriptionally from normal fibroblasts in more than 500 genes encoding proteins such as IGF-2, IL-6, IL-8 and CXCL-1 [10,112]. These CAF produce factors important for the maintenance of stem cell properties of HNSCC cells [14,113]. Production of these molecules by cancer cells and the presence of receptors recognising these molecules are demonstrated in Figure 6. 

CAF also produce large quantities of galectin-1, a very important structural and functional component of ECM, into their microenvironment. This endogenous lectin participates in the transition of fibroblasts to myofibroblasts [29,108]. Galectin-1 also induces apoptosis in T lymphocytes and has an immunosuppressive effect [114]. On the other hand, galectin-1 diminishes resistance of cancer cells to anoikis, which is a typical feature of cancer cells [115]. These observations indicate the pleiotropic effect of galectin-1 in cancer biology. HNSCC with galectin-1-rich stroma host numerous CAF positive for SMA. Tumour cells in the galectin-1-rich environments express genes important for tumour progression such as *SPIN1*, *FUSIP1, TRIM23, PTPLAD1, MAP3K2.*

On the other hand, the galectin-1-rich tumour stroma was not proved as a biomarker of poor patient survival [116,117]. Conversely, low expression of tenascin-C and fibronectin in cancer tissues is typical in low-risk patients, namely in the early stages of the disease [118]. The role of periostin in the stimulation of HNSCC growth and invasion was reported in HNSCC, similarly to other types of tumours [119,120,121].

Similarly to other types of cancer, the HNSCC microenvironment does not markedly differ from healing wounds [37,50], and both share a prominent role of ECM. Data about distinct molecules of ECM as important factors of tissue fibroplastic disorders and HNSCC progression are summarised in Table 1.

In parallel to other malignant diseases, immune cells infiltrating HNSCC represent an essential component of the cancer microenvironment. The presence of CD8^+^ T lymphocytes, Treg and MDSC in tumour specimens or blood samples represents a possible prognostic marker for patients suffering from HNSCC [122,123]. The immune status-based stratification of tumours can be used for further improvement of HNSCC classification [124]. TAM stimulate HNSCC growth, are associated with poor prognosis of patients, and their targeting could represent a potential anti-cancer tool [125].

A remarkable number of HNSCC are caused by HPV infection. Of note, the infiltration of HPV-positive and HPV-negative tumours by immune cells differs [126]. Surprisingly, HPV-induced tumours infiltrated with Treg show improved survival [127]. Furthermore, CD8^+^ T lymphocytes from HPV-positive tumours produce IFN-γ after stimulation and also express PD-1, but not Tim-3. These data indicate that the dual blockade of PD-1 and Tim-3 could be beneficial for HPV-positive HNSCC patients [128].

### Therapeutic Targeting of HNSCC Microenvironment

Modulation of intercellular signalling in the tumour microenvironment can be a valid and robust therapeutic modality. Indeed, it is well recognised that high expression of the VEGF-A factor, which supports tumour vascularisation, is linked to poor prognosis. Combination of anti-VEGF-A humanised monoclonal antibody (bevacizumab) with anti-EGF receptor antibody (cetuximab) can be used for the treatment of recurrent and metastatic HNSCC [135]. Cytokines as prominent mediators of intercellular crosstalk include, e.g., IL-2, IL-6, IL-8 and IFN-α/γ The therapeutic strategies based on the administration of these agents or blocking antibodies were tested in clinical trials or proposed for clinical studies [65,110]; however, with limited success, as reviewed by Schuller and coworkers [136].

The blockade of the immune checkpoints of cell death via PD-1/PD-L1 seems to be more encouraging, similarly to therapy of other types of cancers [137]. Data regarding the results of this therapy were comprehensively summarised by Guidi and coworkers [138]. NK cells, predominantly highly activated ‘super-charged’ NK cells, can be expanded in vitro employing osteoclasts as feeder cells, and their application as cell-based therapeutics can bring a new anti-cancer therapy [139].

Numerous miRNAs are severely dysregulated in cancer including HNSCC, with impact on the intercellular crosstalk between cells of the HNSCC ecosystem. Many of them can be employed as future therapeutic agents [140].

## 5. Conclusions

Similarly to other types of tumours, the microenvironment of head and neck squamous cell carcinoma is formed by a cancer ecosystem. The impact of this ecosystem and its products on the entire organism is shown in Figure 1 and significantly influences the biological properties of tumours and the overall patient’s condition. Cancer-associated fibroblasts and immune cells, as well as their products, can be targeted for therapeutic purposes. Combination of multiple therapeutic targets seems to be beneficial.

## Figures and Tables

**Table 1:** Examples of ECM bioactive molecules supporting growth of HNSCC.

Molecule	Activity
Collagen I	Cancer progression [129]
α1 Chain of collagen XI	Neovascularization, metastasising [130]
Fibronectin isoforms	Cancer progression [131]
Tenascin-C	Cancer progression [132]
Periostin	Stemness maintenance support, cancer progression [120]
Laminin B3	Cancer progression, resistance to actinotherapy [133]
Hyaluronic acid	Cancer progression [134]

## Abbreviations

CAF	Cancer-associated fibroblasts
ECM	Extracellular matrix
HNSCC	Head and neck squamous cell carcinoma
HPV	Human papilloma virus
MDSC	Myeloid-derived suppressor cells
SMA	Smooth muscle actin
TAM	Tumour-associated macrophages
